# Tau protein as a possible marker of cerebrospinal fluid leakage in cerebrospinal fluid rhinorrhoea: A pilot study

**DOI:** 10.11613/BM.2017.030703

**Published:** 2017-08-28

**Authors:** Jean-Baptiste Oudart, Laure Zucchini, François-Xavier Maquart, Xavier Dubernard, Marc Labrousse, Géraldine Fiabane, Alexandra Quedreux, Fabien Litre, Laurent Ramont

**Affiliations:** 1 CHU de Reims, Laboratoire Central de Biochimie, Reims, France; 2 Université de Reims Champagne-Ardenne, UMR CNRS/URCA N°7369, Reims, France; 3 CHU de Reims, Service d’Oto-Rhino-Laryngologie, Reims, France; 4 CHU de Reims, Service de Neurochirurgie, Reims, France; † The authors contributed equally to this paper.

**Keywords:** cerebrospinal fluid, cerebrospinal fluid leakage, cerebrospinal fluid rhinorrhoea, ELISA, Tau protein

## Abstract

**Introduction:**

The management of posttraumatic cerebrospinal fluid (CSF) rhinorrhoea remains a clinical challenge. Cerebrospinal fistula is a dural defect responsible for possible CSF leakage into the contiguous air-filled cavities located at the skull base. The risk of central nervous system infection in these conditions is severe and can be life threatening. Consequently, a specific CSF biomarker might be used in case of difficult diagnosis of CSF rhinorrhoea. CSF Tau protein is a neuronal protein, commonly assessed for diagnosis of Alzheimer Disease (AD). The aim of this study was to determine whether the Tau protein could be a relevant marker of CSF leakage.

**Materials and methods:**

Tau protein measurement was performed by enzyme-linked immunosorbent assay in 13 patients with CSF leakage (CSF rhinorrhoea group), and 8 patients with spontaneous aqueous rhinorrhoea (non-CSF leakage group). The serum concentration of Tau protein was measured by ELISA in both CSF rhinorrhoea group and non-CSF leakage group.

**Results:**

In patients with CSF leakage, CSF Tau protein median concentration was 479 ng/L (197 - 2325 ng/L). On the other hand, the Tau protein concentration was below the lower limit of quantification (LLoQ) (< 87 ng/L) in non-CSF leakage group. Serum Tau protein concentration by ELISA was also below LLoQ (< 87 ng/L) for all subjects.

**Conclusion:**

ELISA measurement of Tau protein in rhinorrhoea fluid may be a reliable and relevant marker for detecting the presence of CSF in the nasal discharge and sign the existence of a CSF leakage.

## Introduction

The diagnosis and management of posttraumatic cerebrospinal fluid (CSF) rhinorrhoea remain a clinical challenge (*1*). Cerebrospinal fistulas (CF) leakage is an abnormal communication between the subarachnoid space and the air-filled cavities of the skull base. The majority of CF is traumatic (head trauma, surgery). They occur preferentially in areas of bone or meningeal weakness (*2*). However, CF can be non-traumatic (idiopathic, congenital malformations, inflammatory processes, tumours, conditions that increase the subarachnoid pressure, slowly eroding the bones of the skull base). The major risk remains central nervous system infection (bacterial meningitis or intracranial abscess) which is potentially fatal, despite therapeutic agents. CF should also be suspected in cases of recurrent infectious complications of central nervous system caused by pyogen bacteria (*2*). The cumulative risk of meningitis was evaluated to 7.4% per week for the first month after injury and exceeds 85% at 10 years follow-up (*3*). In many cases, CF regresses spontaneously.

Nonetheless, a reliable early diagnosis and treatment of CSF leakage is important to prevent infectious complications with appropriate surgery. Currently, there are only few guidelines for the diagnosis of CSF leakage (*4*). The diagnosis is mainly based on clinical features of rhinorrhoea, which can be clear, positional, unilateral or bilateral, intermittent or unnoticed. High resolution computed tomography, magnetic resonance imaging and invasive procedures are necessary in many cases (*5*). Treatment consists of surgical closure of the dural defect. Consequently, it is important to develop biological non-invasive, sensitive, specific and reliable methods for the positive diagnosis of CSF leaks (*5*). These methods could be of great interest when imaging is not contributory or not possible, especially if the patient has contraindications to magnetic resonance imaging (pacemaker, implantable defibrillator, ferromagnetic intraocular foreign body, claustrophobia).

Tau protein, discovered in 1975, is an intraneuronal protein mainly involved in axonal transport and stabilization of microtubules (*6*). It is now well established that an increased concentration of Tau protein in CSF is an important biomarker of Alzheimer disease (AD) (*6*). Tau protein could also be measured in CSF of healthy people. Its concentration is much lower in serum than in CSF (ratio CSF/serum 10:1), so that Tau protein is usually undetectable in other biological fluids with the currently used assay methods (*7*).

The discovery of new biological markers is a challenge for diagnosis and management of posttraumatic CSF rhinorrhoea in three main situations: (i) when additional radiological or interventional examinations do not provide evidence of diagnosis; (ii) when further examination cannot be performed; (iii) in case of discrepancies between clinical features and radiological examinations.

A specific CSF biomarker should be used for difficult diagnosis of CSF rhinorrhoea. Tau protein is commonly assessed in CSF for diagnosis of AD and should be used as a possible specific biomarker of CSF. The aim of this study was to evaluate the diagnostic value of Tau protein as a marker of CF in patients with rhinorrhoea.

## Materials and methods

### Subjects

This study was performed on samples collected after medical prescription of Tau protein assay in the Reims University Hospital (Reims, France). The patients were hospitalized in the Otorhinolaryngology or Neurosurgery department. Patients were included in this study from October 2011 to December 2016. Three groups of patients were assessed. The CSF rhinorrhoea group included 13 patients with rhinorrhoea due to CF leakage (7 females and 6 males; median age 59 years (27-79); 8 post chirurgical CF leakages and 5 posttraumatic CF leakages). The diagnosis of CSF rhinorrhoea was confirmed after surgery exploration, associated in some cases with sodium-fluorescein detection. The non-CSF leakage group included 8 patients with spontaneous aqueous rhinorrhoea due to viral or allergic rhinopathy (5 women and 3 men; median age 31 years (18-47)). These patients never presented any clinical feature of dural defect, head trauma, meningitis, or meningeal weakness. The sera group included the sera of ten patients from CSF rhinorrhoea group, three patients of non-CSF leakage group and three other healthy control patients (7 women and 3 men; median age 49 years (18-79)). It is therefore important to assay in parallel Tau protein in the serum and in the rhinorrhoea fluid using the same technique. For each patient, rhinorrhoea fluid and serum Tau protein concentrations must be assessed in the same way to avoid any influence of blood contamination of rhinorrhoea sample.

### Methods

All nasal discharge (400 μL) were collected, stored and processed in 5 mL polypropylene tubes without additive (ref 62.610.201, Sarstedt, Marnay, France). These tubes do not adsorb the Tau protein and thus do not affect results (*8*). They were centrifuged for 10 minutes at 2000xg at 4 °C and 300 µL of the supernatant was collected in another tube for Tau protein assay and immediately stored at - 80 °C until use. Tau protein concentrations in CSF remains stable at least for one year when the sample is stored at - 80 °C (*9*).

Concentration of Tau protein was measured in the three groups by a sandwich Enzyme Linked ImmunoSorbent Assay (ELISA) (INNOTEST hTau Ag, Fujirebio Europe, Ghent, Belgium) according to manufacturer’s instructions. This assay was able to quantify total Tau protein, including its six isoforms. For each kit, we used the lower limit of quantification (LLoQ) according to the manufacturer’s instruction. LLoQ may vary between 34 to 87 ng/L depending on the kit used in the study. In case of concentrations higher than the limit of quantification (about 1000 ng/L) the samples were diluted. According to the manufacturer recommendations, the reference values for the Tau protein in the cerebrospinal fluid measured by ELISA are as follows: 21 to 51 years: 136 ± 89 ng/L, 51 to 70 years: 243 ± 127 ng/L, > 70 years: 341 ± 171 ng/L.

The analysis was blind to clinical data and performed when required by the clinician. Tau protein measurement was assessed after medical prescription and performed routinely in our laboratory. Our laboratory is involved in external quality control program from Alzheimer’s association. No additional examinations were carried out and there were no risk or constraint for the patient. Approbation by an institutional/national ethics committee is not necessary in this case.

### Statistical analysis

The results obtained were analysed using StatEL software (Ad Science, Paris, France). Comparison between groups was done with Mann-Whitney nonparametric test and P values less than 0.05 were considered statistically significant. Cut-off for the best sensibility and specificity of the marker was determined with a ROC Curve.

## Results

Tau protein concentration was detected in all patients in CSF rhinorrhoea group. Its mean concentration was 711 ng/L ranging from 197 ng/L to 2325 ng/L (median concentration 479 ng/L). By contrast, Tau protein was below the LLoQ of the ELISA assay (< 87 ng/L) in all samples from non-CSF leakage group. In all cases, the serum concentration of the Tau protein was also below the LLoQ (< 87 ng/L). The concentration of Tau protein was significantly higher in the CSF rhinorrhoea group than in non-CSF leakage group (P < 0.001).

According to these results, a cut-off of 87 ng/L was proposed to discriminate the CSF rhinorrhoea group and non-CSF leakage group. With this cut-off, sensitivity (100%) and specificity (100%) of Tau protein measurement by ELISA were excellent since Tau protein was present at detectable concentration in 100% of CSF rhinorrhoea *vs* 0% in the non-CSF leakage group.

## Discussion

In this study, we demonstrate that Tau protein measurement in the rhinorrhoea fluid may be a relevant new marker for the diagnosis of CSF leakage with a good sensitivity (100%) and good specificity (100%). Tau protein was higher in the CSF rhinorrhoea group compared to non-CSF leakage group.

Diagnosis of skull base defects, either traumatic or not, is sometimes difficult. Laboratory tests are then of great interest. Several biological methods have been proposed to differentiate CSF rhinorrhoea from other spontaneous rhinorrhoea (for instance, linked to respiratory infections, tears or blood contamination).

Biological investigation of a suspected rhinorrhoea consisted for a long time in glucose content determination, using glucose oxidase strips. This screening test has the advantage of being easily done in an emergency context and at the bedside. It is fast, inexpensive and widely available. However, it has a poor sensitivity and specificity with high potential of misdiagnosis (*2*, *4*). Moreover, contamination by blood, even in small quantities, can lead to false positive results or misinterpretation. These restrictions make this test very difficult to interpret and it should be abandoned in favour of more reliable methods. However, an algorithm was proposed to reinstate its use (*9*).

β2-transferrin and β-trace protein (lipocalin-like prostaglandin D or L-PGDS) have also been proposed for the diagnosis of CF (*10*).

β2-transferrin is a desialylated isoform of transferrin, which is absent in nasal, lachrymal and mucous secretions (*2*). Several detection techniques are possible, mainly immunofixation electrophoresis (IFE) and isoelectric focusing (*11*). However, these two techniques can be disrupted by the presence of blood in the sample.

β-trace protein (prostaglandin D2 synthase), a member of the family of lipocalins, is the second most abundant protein in CSF after albumin (*12*, *13*). β-trace protein is also measurable in healthy subject’s rhinorrhoea fluid. It appeared as a good, reliable, reproducible biomarker and was more sensitive than β2-transferrin for the diagnosis of CSF rhinorrhoea (*4*). However, the main limitation of the use of β-trace protein as biomarker of CSF rhinorrhoea is the cut-off value which ranges from 0.25 to 6 mg/L according to the literature (*14*). A cut-off of 1.11 mg/L was determined by Risch *et al*., with 100% specificity and 93% sensitivity (*15*).

The technique used in this study does not allow the detection of serum or nasal secretion Tau protein. Consequently, Tau protein was not detected in non-CSF leakage group whereas it was detected in the case of CSF leakage. The reference values of Tau protein in CSF were comparable to those found in the CSF rhinorrhoea group. Despite the small number of samples in each group, sensitivity and specificity of Tau protein measurement by ELISA were excellent since Tau protein was present at high concentration in 100% of CSF rhinorrhoea vs 0% in non-CSF leakage group. Tau concentrations did not appear to be associated with any of the demographic or clinical characteristics of patients with CSF rhinorrhoea. Tau protein measurement seems more efficient than other biomarkers commonly used to diagnose CSF leakage. Contrarily to the previous markers, it was not influenced by blood contamination. Blood contamination remains the main confusing factor, which could lead to misinterpretation of the currently used biomarkers. In all cases, the serum concentration of Tau protein was below the LLoQ of the assay. Our results demonstrate that an increased concentration of Tau protein in rhinorrhoea was only caused by CSF leakage. This is a great advantage since blood contamination often occurs after head trauma. Surgery, head trauma or meningitis are often associated with limited central nervous system cell lysis. Tau protein measurement is commonly used in CSF as a marker of neuronal lysis, which could explain the highest concentrations, measured in some rhinorrhoea samples. This higher concentration of Tau protein in CSF in case of cell lysis may contribute to the sensitivity of Tau protein measurement in CSF rhinorrhoea and overcome the false negative β2 transferrin detection reported in the case of *Streptococcus pneumoniae* infection as well as the decreased β-trace protein concentration reported in meningitis (*4*, *16*).

By contrast, Tau protein is lower in case of normal pressure hydrocephalus, which could lead to false negative results (*17*). To avoid the risk of confusing results, a blood sample could be assessed in association with rhinorrhoea sample. Blood contamination remains the main confusing factor, which could lead to misinterpretation of biological results (*i.e*. false increased concentration of the β trace protein in rhinorrhoea). Tau protein must be assessed in both serum and rhinorrhoea fluid, using the same technique. According to the ELISA assay used in this study, the serum concentration of Tau protein was below the LLoQ of the assay, which demonstrate that blood contamination did not influence the rhinorrhoea of Tau protein. ELISA has also the advantage to require a low sample volume (50 μL). This is an important point, given the low volumes available for these types of samples. The main disadvantages of the ELISA assay is the high number of patients needed to completely fill the plate (cost of the test) and the duration of the assay (first incubation lasts overnight). Moreover, for each run, duplicate wells for calibrators, controls, and samples are needed. Thus, a major limitation of ELISA is the fact that it is not suitable as a rapid and emergency assay. The development of automated assay could overcome this limitation.

In conclusion, determination of Tau protein by ELISA uses well standardized steps or the sample handling procedure, which permits to obtain reproducible results. It is widely used in the diagnosis of AD although internationally recognized reference material is not yet available. This pilot study is encouraging and suggests that, in the future, it could be easily performed routinely and allows reliable detection of CSF in the case of rhinorrhoea related to a CSF fistula. Contamination by blood does not confound the results since serum Tau protein was below the LLoQ with this method for all samples. Our study shows that Tau protein measurement in the rhinorrhoea fluid may be a relevant new marker for the diagnosis of CSF leakage.
